# The effect of amphetamine-type stimulants on immune response in HIV-infected individuals: a retrospective cohort study

**DOI:** 10.3389/fimmu.2026.1775159

**Published:** 2026-02-09

**Authors:** Tao Li, Zuoliang Li, Hai Liu, Jinsi Chen, Yanwei Li, Xin Zhou, Su Zhang, Bei Li, Yulan Ren

**Affiliations:** 1 TCM-WM Department, The Second Affiliated Hospital of Chongqing Medical University, Chongqing, China; 2 Chongqing Key Laboratory of Integrative Oncology in Traditional Chinese Medicine, Chongqing, China; 3 School of Acupuncture and Tuina, Chengdu University of Traditional Chinese Medicine, Chengdu, China; 4 AIDS Antiviral Treatment Office, Ziyang Rehabilitation Center for Drug Addiction, Ziyang, China; 5 AIDS Antiviral Treatment Office, Ziyang Yanjiang District People’s Hospital, Ziyang, China; 6 Cardiology Department, Affiliated Hospital of Chengdu University of Traditional Chinese Medicine, Chengdu, China; 7 School of Chinese Classics, Chengdu University of Traditional Chinese Medicine, Chengdu, China

**Keywords:** amphetamine-type stimulants, antiretroviral therapy, cohort study, HIV, immune response

## Abstract

**Background:**

The co-occurrence of amphetamine-type stimulant (ATS) abuse and HIV infection is prevalent, yet the impact of ATS abuse history on immune reconstitution in people living with HIV (PLWH) remains uncertain. We aim to assess the impact of ATS abuse history on immune reconstitution in PLWH with low CD4+ levels and high ART adherence.

**Methods:**

Using data from China’s National Free Antiretroviral Treatment Program and Drug Rehabilitation Management Platform data, we identified PLWH who met the inclusion criteria between 2016 and 2022. Propensity score matching paired participants. Primary outcomes focused on immune response after 2 years of ART, and secondary outcomes including CD4+ T-cell count recovery, net increase in CD4+ T-cell count, CD4+ T-cell growth rate, time to immune response, and AIDS-related mortality.

**Results:**

After matching 2,372 PLWH into ATS and non-ATS cohorts, the ATS group exhibited lower immune response rates, delayed responses, smaller increases in CD4+ T-cell counts, and slower initial CD4+ T-cell growth, although growth rates were comparable at later time points. Cox regression analysis identified ATS exposure as an independent risk factor for poorer immune responses 2 years after ART initiation [HR: 0.558 (0.485-0.643), P<0.001]. Kaplan-Meier analysis demonstrated a lower cumulative immune response rate in the ATS group. Longer ATS exposure was associated with reduced immune response rates in PLWH after ART initiation.

**Conclusions:**

A history of ATS abuse hinders HIV immune reconstitution, underscoring the need for tailored services for PLWH with ATS exposure.

## Background

The global prevalence of HIV/AIDS continues to pose a significant public health challenge. As of 2023, the number of people living with HIV (PLWH) is approximately 39 million (1). The widespread promotion of antiretroviral therapy (ART) has markedly prolonged patient survival. Nevertheless, around 20%-30% of patients encounter incomplete immune reconstitution, characterized by delayed or persistently low CD4+ T-cell recovery (2). This condition is associated with a significant increase in the incidence of opportunistic infections and mortality (3–5). Previous studies have identified the effects of factors such as age (6), baseline CD4 levels (7), and co-infections (e.g., hepatitis C) (8) on immune response. However, the contribution of behavioral factors, such as substance use disorders, has not been fully clarified.

Since the beginning of the 21st century, synthetic drugs represented by amphetamine - type stimulants (ATS) have gradually become the most widely abused and rapidly spreading drugs globally (9). ATS use disorders, typified by methamphetamine and ecstasy, has further complicated HIV prevention and control (10). ATS use is closely associated with high-risk sexual behavior (9) and needle sharing (11). and ATS use disorders can reduce ART adherence and negatively affect immune responses in PLWH (12, 13). Numerous studies have confirmed the close association between ATS exposure and immune suppression (14, 15). Animal experiments have demonstrated that the number of T cells in mice injected with amphetamine over a prolonged period is significantly reduced (16), potentially due to ATS-induced apoptosis of thymic and splenic lymphocytes (17). Cellular studies have shown that ATS exposure induces mitochondrial oxidative damage and dysfunction in primary human T cells (18). Moreover, ATS use disorders may increase susceptibility to HIV by activating CD4+ T-cell (19, 20). Related studies have reported that PLWH with ATS use disorders have higher viral loads than non-users (21, 22). Currently, it remains unclear whether a history of ATS use disorders affects the immune response among PLWH with good adherence after ART initiation.

This study aims to explore whether a history of ATS use disorders independently affects the immune response among PLWH after initiating ART. By matching potential confounders, we hypothesize that PLWH with a history of ATS use disorders have lower CD4+ T-cell recovery rates and immune response rates, which may be associated with the duration of the ATS use disorders. The research findings may provide evidence-based support for individualized management of ATS use disorders.

## Methods

### Data source

The research data were derived from clinical records in two databases:

The database of China’s National Free Antiretroviral Treatment Program (NFATP) subject to routine data entry and verification by personnel from the Chinese Center for Disease Control and Prevention. This database supports the long-term, ongoing collection of follow-up data, treatment information, and mortality data for PLWH reported over the years.

The Drug Rehabilitation Management Platform (DRMP) of the Drug Rehabilitation Administration is legally registered and supervised by law enforcement officers at drug rehabilitation centers. Individuals with ATS use disorders are required to undergo 2 years of drug rehabilitation treatment. The DRMP collects demographic information, history of drug use disorders, medical examination reports, and data on communicable diseases among individuals with ATS use disorders. For PLWH, the Antiviral Therapy Office provides free antiretroviral therapy. Medical personnel are responsible for distributing ART drugs and supervising patients’ use. Over a 2-year period, PLWH initiating ART undergo routine monitoring of CD4+ T-cell counts, viral load, and drug resistance testing to ensure compliance with treatment and follow-up.

During data collection, paper records were used to verify the data.

### Patient population

Since June 2016, China has implemented universal free antiretroviral treatment for PLWH, regardless of CD4+ T-cell count or clinical stage (23). The revision of treatment criteria has significantly expanded treatment coverage. Consequently, this study enrolled PLWH who newly initiating ART between August 1, 2016, and July 31, 2022.

### Exposure status

We accessed the DRMP of the Drug Rehabilitation Administration, filtered records for drugs categorized as ATS, and identified individuals who commenced ART for the first time. Patients with a history of multiple drug use disorders were excluded. Only patients with ATS use disorders were classified as the exposed group.

We retrieved data from the NFATP database for individuals who were newly registered for ART. Patients who acquired HIV through injection drug use were excluded. Patients without a history of drug use disorders (all drugs under national control) were classified as the non-exposed group.

### Inclusion criteria

Age 18–65 years;
Initiated ART between August 1, 2016, and July 31, 2023;
3.HIV/AIDS diagnosis confirmed by an official diagnosis report issued by the Center for Disease Control and Prevention;
ART-naïve individuals with a baseline CD4+ T-cell count ≤350cells/mm^3^ and at least one documented viral load result;
For the exposed group, a confirmed ATS urine test result was required; for the non-exposed group, the HIV transmission route excluded injection drug use, and individuals had no history of drug use disorders.

### Exclusion criteria

1. Presence of severe opportunistic infections, malignant tumors, and end-stage organ failure;
2. Incomplete baseline covariate data in medical records;
3. Abnormal routine blood routine, liver function, or kidney function;
4. A history of multiple drug use disorders in the exposed group.

### Follow-up and data collection

Both cohorts were followed for 2 years. CD4+ T-cell counts were assessed every 3 months after ART initiation, for a total of 8 measurements. Viral load was monitored at 1 and 3 months after ART initiation and subsequently every 3 months until viral load suppression was achieved. Death attributable to opportunistic infections, malignancies, or other AIDS-related causes was classified as immune response failure. We recorded essential demographic data (gender, age, BMI), HIV-related medical history (date of diagnosis, route of HIV infection, CD4+ T-cell count, viral load, WHO stage, ART initiation date, and ART scheme), and information on communicable diseases (syphilis, tuberculosis, hepatitis B, hepatitis C). Additionally, for the exposed cohort, we collected details on ATS abuse (drug name and duration of use).

### Outcome

The primary outcome was the immune response. Given the lack of a standardized definition for “immune response”, based on insights from prior research (24–26), the immune response in this study was defined as meeting all three of the following criteria: (1) two consecutive undetectable HIV-1 RNA measurements (viral load <50 copies/mL); (2) CD4 counts consistently >350 cells/μL on two consecutive measurements; and (3) a minimum interval of at least 1 month between the two assessments.

Secondary outcomes included time to achieve immune response, CD4+ T-cell growth rate, net increase in CD4+ T-cell count, immune response failure rate, and CD4+ T-cell count recovery. Based on prior research (27), CD4+ T-cell count recovery was defined as achieving two consecutive CD4+ T-cell counts ≥500 cells/mm^3^ with a viral load <50 copies/mL, with a minimum interval of at least 1 month between evaluations.

### Covariates

1. Demographic characteristics: gender, baseline age, BMI (28).
2. Other communicable diseases: hepatitis B, hepatitis C, syphilis, tuberculosis (29–32).
3. HIV-related clinical history: time of HIV diagnosis, route of HIV infection; time of ART initiation, ART scheme, baseline CD4 count, viral load, WHO stage (33–35).

### Statistical analysis

To address missing follow-up data (**Supplementary Table 1**), we used complete baseline information as auxiliary variables and applied the Markov Chain Monte Carlo (MCMC) method to impute missing data (36). Given missing data rates ranging from 5% to 15%, we conducted 20 imputations to account for missingness (37). Univariate analysis was performed across the 20 imputed datasets, yielding consistent results before and after multiple imputation. Using Rubin’s rules to combine estimates from the 20 imputed datasets, we generated a pooled dataset for subsequent statistical analyses. This study employed Propensity Score Matched (PSM) analysis. All covariates were treated as potential confounders, and 1:1 matching between the exposed and non-exposed groups was performed using the nearest-neighbor caliper matching with a caliper of 0.02.

Statistical analyses were conducted using SPSS v25.0 and R v4.1.3, with P<0.05 considered statistically significant. Differences in baseline characteristics and clinical outcome indicators between groups were assessed using the Pearson chi-square test (for categorical variables) and the Kruskal-Wallis test (for continuous variables). Univariate and multivariate analyses were conducted using the Cox proportional hazards regression model to examine associations between baseline variables and immune response. The Kaplan-Meier method was used to compare cumulative immune response rates between the two groups. Subgroup analyses were performed in the exposed and non-exposed cohorts. Furthermore, a dose-response analysis was conducted to evaluate the effect of the duration of ATS use disorders on immune response.

## Results

### Baseline characteristic

**Figure 1:**
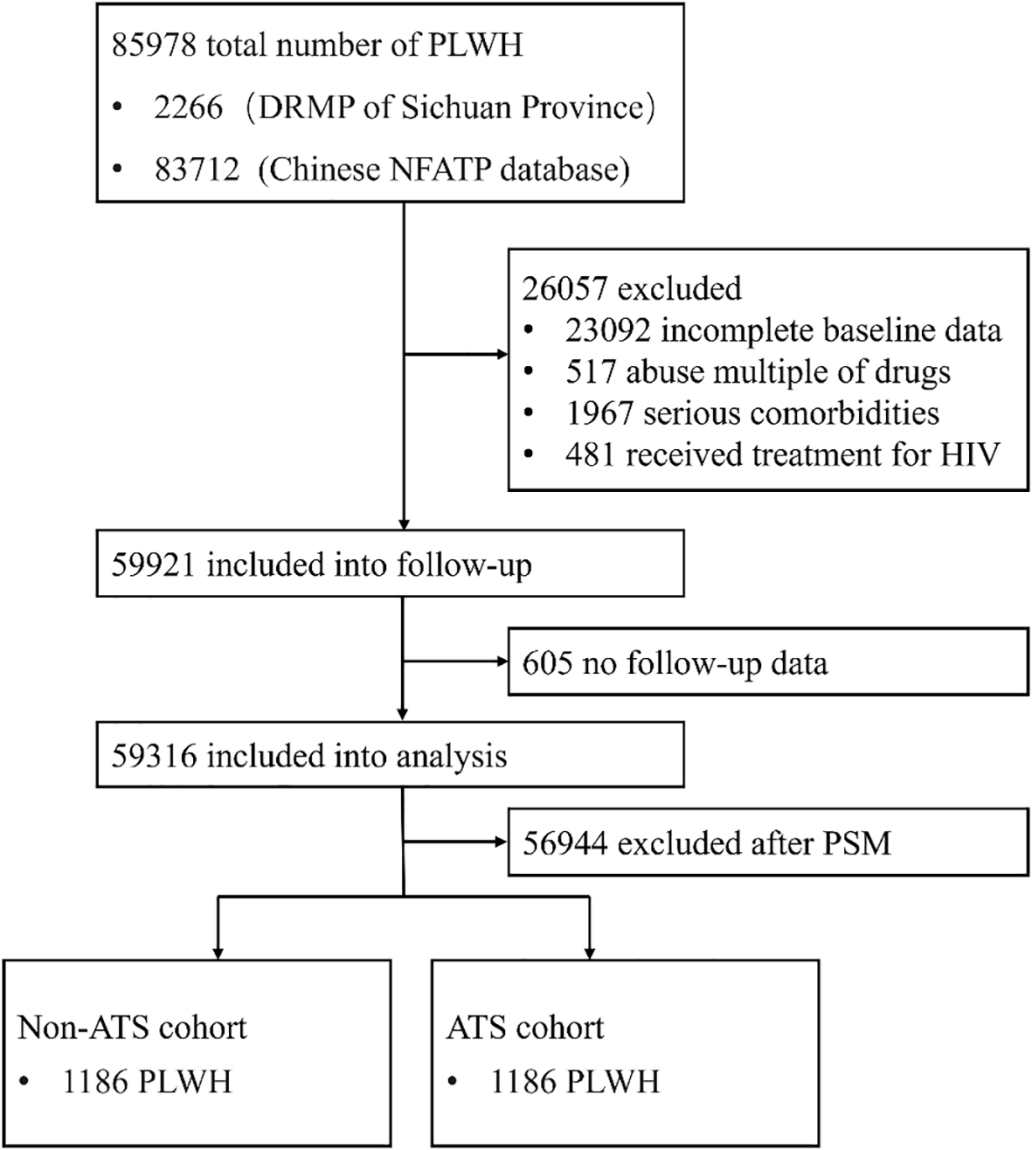
The inclusion and exclusion process of PLWH in this study; PLWH, people living with HIV; DRMP, Drug Rehabilitation Management Platform; NFATP, National Free Antiretroviral Treatment Program; PSM, propensity scores matching; ATS, amphetamine-type stimulant.

2,266 PLWH with a history of ATS use disorders were identified as initiating ART for the first time. After excluding patients with incomplete baseline data, severe comorbidities, and a history of multiple drug use disorders, 1,372 PLWH were follow-up. Subsequently, 16 patients with no follow-up records were excluded, resulting in 1,356 exposed patients included for PSM. Moreover, in the NFATP database, 83,712 HIV-infected individuals were screened, and 58,549 patients who met the inclusion criteria were included in the follow-up. After excluding 589 patients with no follow-up records, a final cohort of 57960 PLWH was retained for analysis (**Figure 1**).

### Propensity scores matching

Before PSM, baseline covariates differed between the two patient groups (**Supplementary Table 2**). After PSM, 1,186 cases were successfully matched between the two cohorts. Notably, after PSM, most potential confounding factors were no longer statistically significant, except for viral load, the interval between HIV diagnosis and ART initiation, and WHO stage.

### Immune response

Among the 2,372 PLWH after PSM, 2,285 patients completed the 2-year follow-up, and 87 deaths were recorded during this period (**Supplementary Table 3**). Over a total follow-up of 4,655.5 person-years, 1,002 patients achieved immune response, yielding an overall incidence density of 21.52 per 100 person-years. In the exposed cohort, 1,186 individuals were followed up for a cumulative 2,315.5 person-years, with 454 patients achieving immune response, corresponding to an immune response density of 19.61 per 100 person-years. In comparison, 1,186 patients in the non-exposed cohort were followed for 2,340 person-years, with 548 individuals attaining immune response during ART, resulting in an immune response density of 23.42 per 100 person-years.

**Figure 2:**
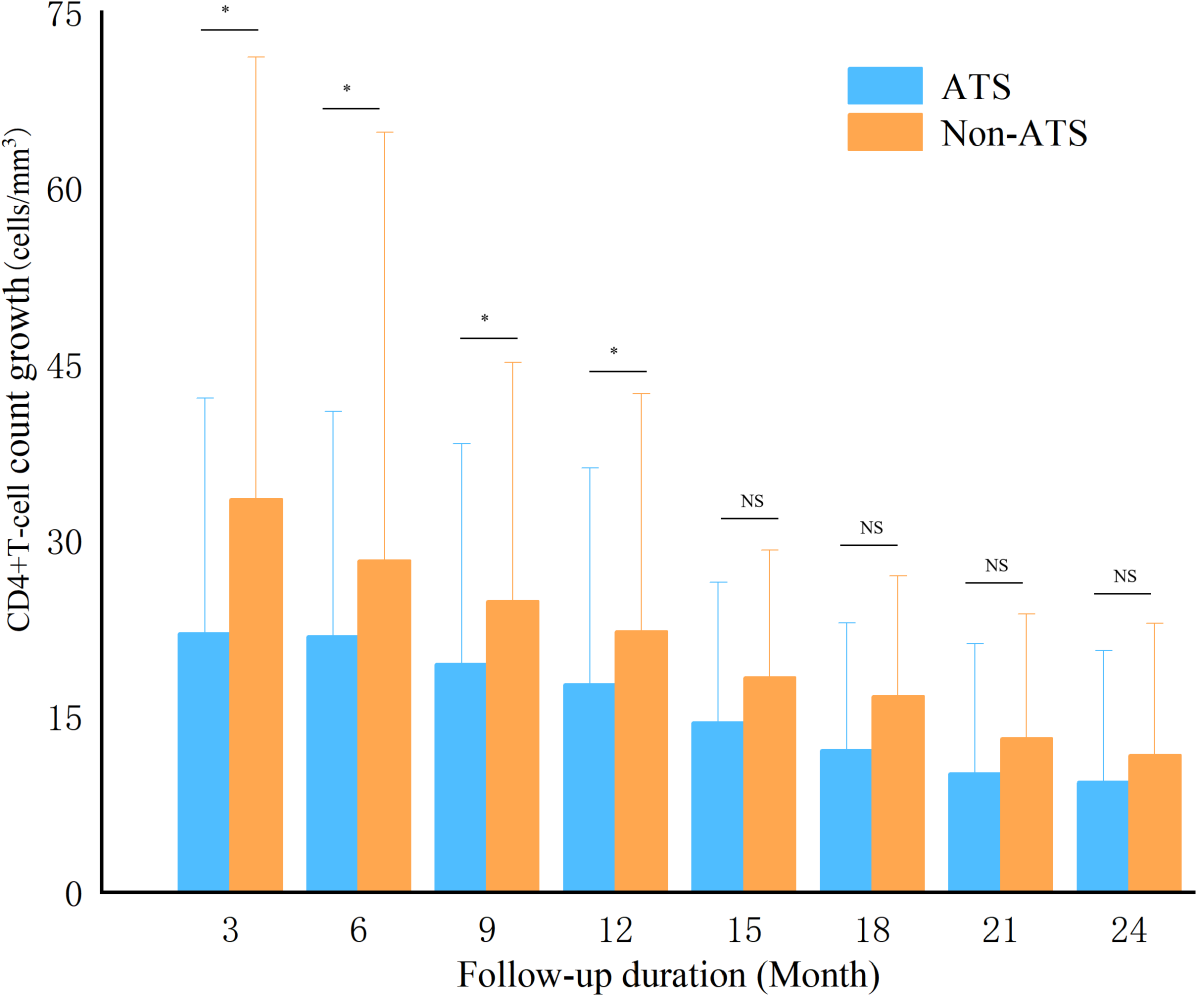
Growth of CD4+T cells during follow-up. Using Kruskal Wallis test, the exposed and non exposed groups were compared at each time point, * represents P<0.05, NS represents P>0.05; P<0.05 indicating statistical differences between the groups; ATS, amphetamine-type stimulant.

During the follow-up period, a total of 116 patients achieved CD4+ T-cell count recovery, yielding an incidence density of 24.92 per 1000 person-years. Among these, 29 individuals in the exposed cohort achieved CD4+ T-cell count recovery, with an incidence density of 12.52 per 1000 person-years, while 87 individuals in the non-exposed cohort achieved CD4+ T-cell count recovery, corresponding to an incidence density of 37.18 per 1000 person-years. The time to immune response was shorter in the non-exposed cohort (14.50 ± 7.30 months) than in the exposed cohort (16.62 ± 7.14 months) (**Table 1**). The immune response failure rate was higher in the exposed cohort(4.47%) than in the non-exposed cohort(2.02%). After ART initiation, the non-exposed cohort exhibited more rapid early increases in CD4+ T-cell counts during the first 12 months, with growth rates becoming comparable between the two cohorts over the subsequent 12 months. After 2 years of follow-up, the net increase in CD4+ T-cell count was significantly greater in the non-exposed cohort (167.99 ± 89.11 cells/mm^3^) than in the exposed cohort(119.44 ± 88.13 cells/mm^3^) (**Figure 2**).

**Table 1:** Immune reconstitution of PLWH in ATS and Non-ATS.

Outcome	ATS	Non-ATS	P
Baseline CD4+ T-cell (cells/mm^3^)	161(106,221)	159(99,221)	0.643
Immune response time (month)	16.62 ± 7.14	14.50 ± 7.30	<0.001
AIDS-related mortality rate (%)	4.47	2.02	0.001
Net increase in CD4+ T-cell (cells/mm^3^)	119.44 ± 88.13	167.99 ± 89.11	<0.001

P<0.05 indicating statistical differences between the groups; ATS, amphetamine-type stimulant.

### Risk factors related to immune response

In the multivariate Cox regression analysis, variables with P < 0.05 in the univariate Cox regression were included (**Table 2**). The results showed that the exposed cohort had a lower 2-year immune response rate than the non-ATS cohort. A history of ATS exposure was identified as an independent risk factor for immune response among PLWH.

**Table 2:** Cox regression analyses of PLWH immune response.

Variable	Univariate Cox regression analysis			Multivariable Cox regression analyses		
	HR	95%CI	P	HR	95%CI	P
Gender						
Male	Ref.					
Female	1.128	0.949-1.342	0.171			
Age						
18-29	Ref.			Ref.		
30-39	0.714	0.616-0.828	**<0.001**	0.758	0.650-0.883	**<0.001**
40-49	0.533	0.413-0.688	**<0.001**	0.543	0.412-0.717	**<0.001**
50-65	0.274	0.191-0.393	**<0.001**	0.311	0.195-0.498	**<0.001**
Route of infection						
Heterosexual	Ref.					
Homosexual	1.159	0.997-1.343	0.054			
Blood	1.057	0.677-1.649	0.808			
Mother-baby	1.459	0.843-2.527	0.177			
Unclear	1.144	0.921-1.422	0.224			
BMI						
<18	Ref.			Ref.		
18-25	2.828	2.379-3.363	**<0.001**	3.043	2.541-3.644	**<0.001**
>25	2.308	1.745-3.054	**<0.001**	6.811	4.933-9.404	**<0.001**
WHO stage						
I	Ref.			Ref.		
II	0.690	0.563-0.847	**<0.001**	0.730	0.582-0.915	**0.006**
III	0.463	0.363-0.590	**<0.001**	0.741	0.553-0.992	**0.044**
IV	0.131	0.065-0.263	**<0.001**	0.401	0.179-0.898	**0.026**
Time interval^a^						
0-7	Ref.			Ref.		
8-30	0.822	0.709-0.953	**0.009**	0.815	0.702-0.946	**0.007**
31-90	0.716	0.586-0.876	**0.001**	0.750	0.600-0.938	**0.012**
91-365	0.390	0.310-0.489	**<0.001**	0.577	0.431-0.773	**<0.001**
>365	0.236	0.154-0.360	**<0.001**	0.390	0.215-0.706	**0.002**
Viral load						
0-5000	Ref.			Ref.		
5001-30000	0.711	0.549-0.920	**0.010**	0.749	0.574-0.977	**0.033**
30001-100000	0.698	0.544-0.894	**0.004**	0.714	0.552-0.923	**0.010**
100001-500000	0.369	0.280-0.486	**<0.001**	0.550	0.406-0.745	**<0.001**
>500000	0.319	0.238-0.428	**<0.001**	0.514	0.368-0.719	**<0.001**
CD4+ T-cell count						
0-100	Ref.			Ref.		
101-200	3.300	2.587-4.211	**<0.001**	2.491	1.902-3.263	**<0.001**
201-350	9.842	7.722-12.544	**<0.001**	10.397	7.956-13.586	**<0.001**
Initial ART scheme						
EFV+3TC+TDF	Ref.					
3TC+AZT+NVP	0.817	0.643-1.039	0.100			
3TC+AZT+EFV	0.972	0.746-1.266	0.832			
Other	1.126	0.915-1.386	0.262			
Syphilis						
No	Ref.			Ref.		
Yes	0.552	0.447-0.682	**<0.001**	0.785	0.634-0.973	**0.027**
Tuberculosis						
No	Ref.			Ref.		
Yes	0.680	0.536-0.863	**0.001**	0.603	0.473-0.769	**<0.001**
Hepatitis B						
No	Ref.			Ref.		
Yes	0.553	0.430-0.709	**<0.001**	0.650	0.504-0.837	**0.001**
Hepatitis C						
No	Ref.			Ref.		
Yes	0.214	0.107-0.429	**<0.001**	0.475	0.234-0.961	**0.038**
Abuse of ATS						
No	Ref.			Ref.		
Yes	0.725	0.640-0.821	**<0.001**	0.558	0.485-0.643	**<0.001**

^a^The interval between HIV diagnosis and initiation of ART. P<0.05 indicating statistical differences between the groups; ATS, amphetamine-type stimulant.

Bold values represent statistical differences.

### K-M curve of cumulative immune response

**Figure 3:**
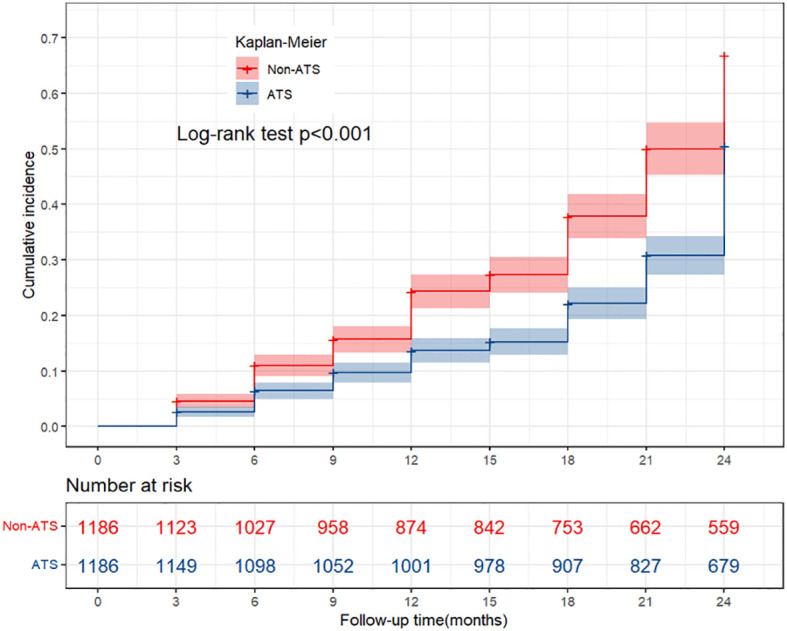
The K-M curve of cumulative immune response. Using the Log rank test, compare the cumulative immune response between the exposed and non exposed groups during the follow-up period. P<0.05 indicating statistical differences between the groups; ATS, amphetamine-type stimulant.

Kaplan–Meier analysis of cumulative immune response showed that PLWH without a history of ATS use disorders had a higher cumulative immune response rate after ART initiation than those with such a history. The cumulative immune response rate as significantly lower in the exposed group (50.86%) than in the non-exposed group (67.14%) (**Figure 3**).

### Subgroup analysis

**Figure 4:**
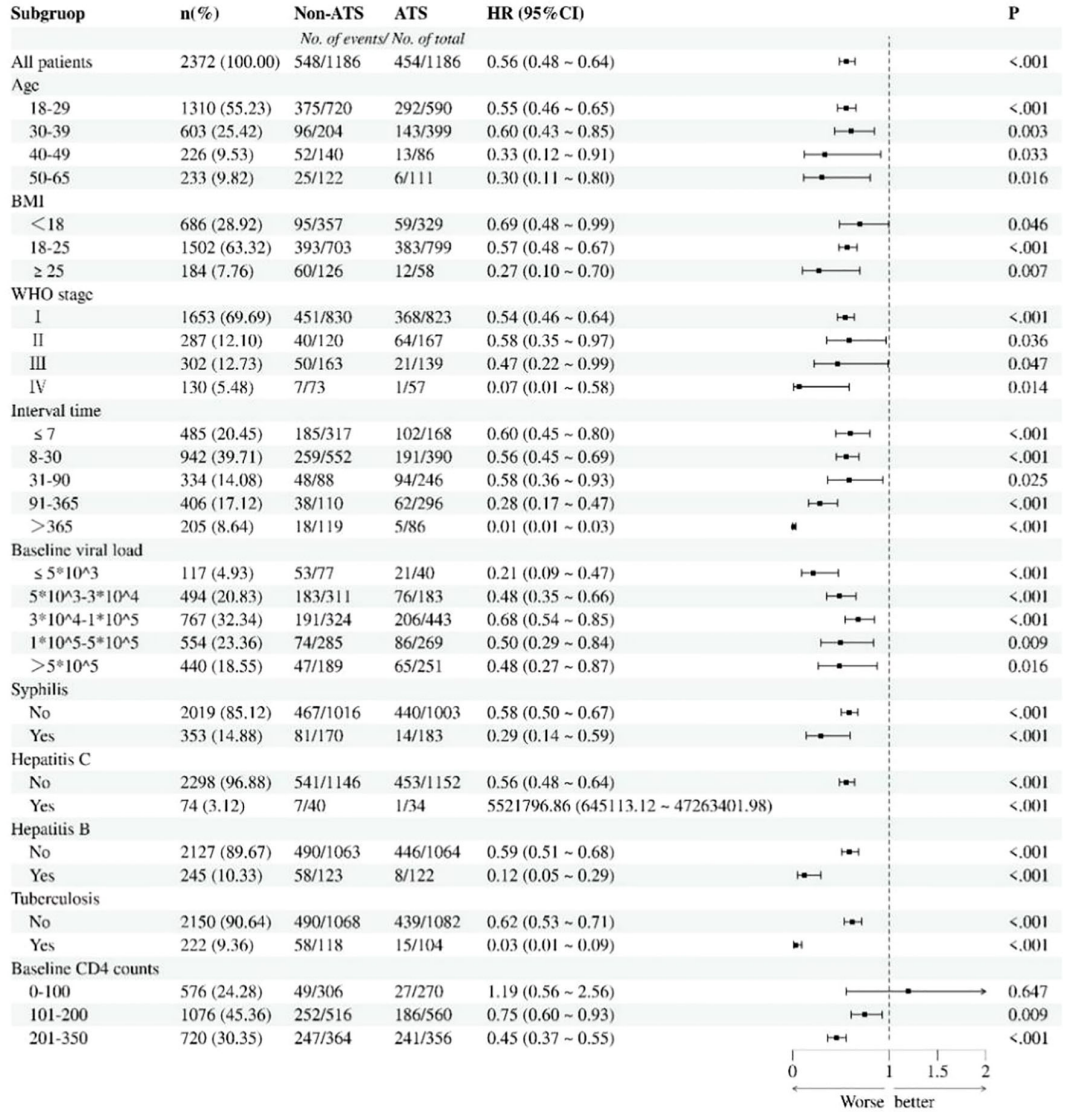
Subgroup analysis of immune response in ATS and non-ATS cohorts. Risk ratios were adjusted based on age, BMI, WHO stage, interval time, baseline viral load, baseline CD4 count, infectious disease, and ATS exposure.Using Pearson chi square test, compare the hazard ratios between the exposed and non exposed groups within each subgroup. P<0.01 indicates statistical differences between groups. ATS, amphetamine-type stimulant.

Subgroup analyses were performed according to potential risk factors influencing immune response. The findings showed that, except for the subgroup with baseline CD4+ T-cell counts of 0–100 cells/mm^3^, a history of ATS use disorders remained as a risk factor for immune response among PLWH across all other subgroups (**Figure 4**).

### The dose-response relationship between exposure time and immune response

**Figure 5:**
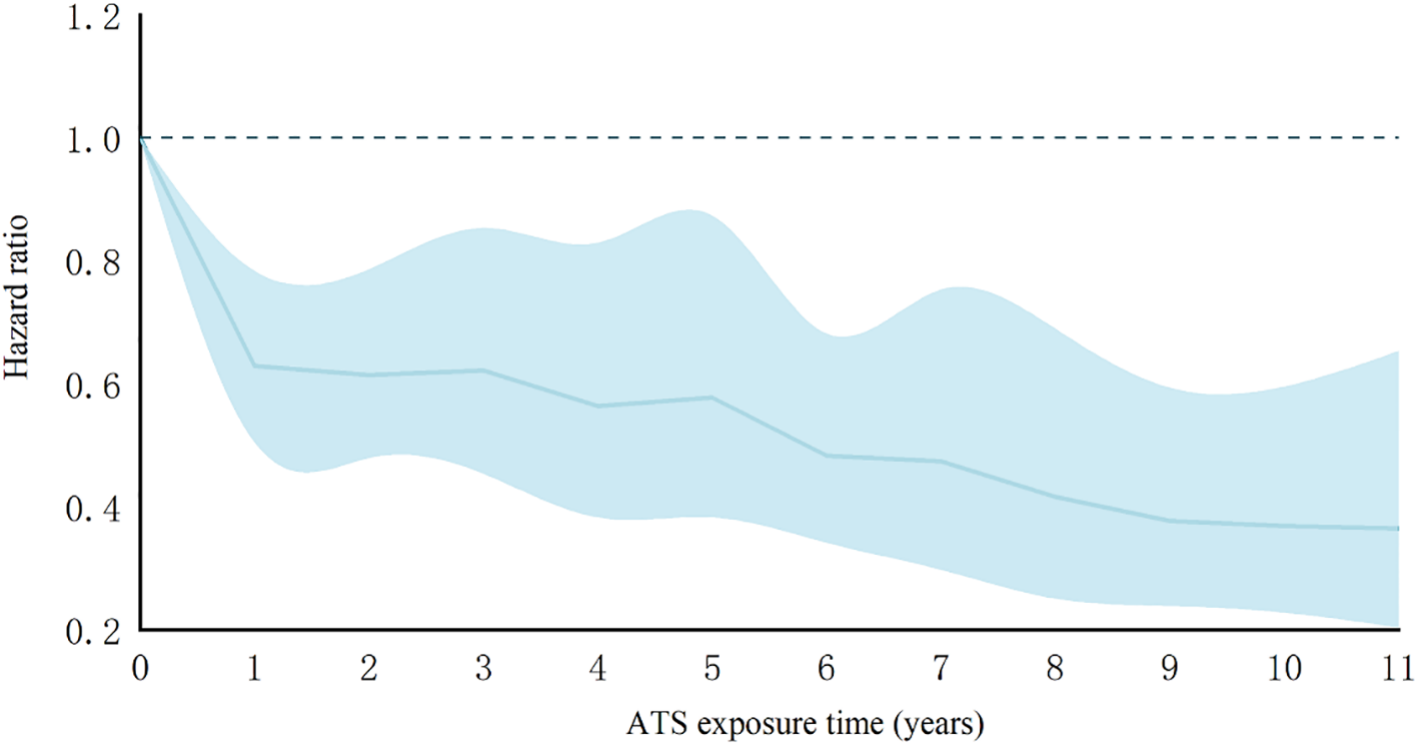
Dose-response relationship between ATS exposure level and immune response rate in PLWH patients; Shaded areas show 95% CIs. Risk ratios were adjusted based on age, BMI, WHO stage, interval time, baseline viral load, baseline CD4 count, and infectious disease. Use Cox proportional hazards regression model.

Among the 1,186 individuals with a history of exposure, 1,035 patients reported a median ATS abuse duration of 3.38 years (1.18, 6.78) prior to ART initiation. Dose-response analysis showed that the association between a history of ATS use disorders and immune response among PLWH was weaker at 1 year of exposure [HR: 0.629 (0.505-0.783)] than at 11 years of exposure[HR: 0.365 (0.204-0.653)](**Figure 5**).

## Discussion

To our knowledge, comprehensive investigations assessing how prior ATS use disorders affect immune response in PLWH remain limited. In our study, the ATS-exposed cohort was younger at enrollment, had longer intervals between HIV diagnosis and ART initiation, and showed higher rates of communicable diseases than the non-exposed cohort. These findings are consistent with established epidemiological patterns: ATS use disorders predominantly affect younger populations (38, 39), whereas aging is a prominent feature of the broader HIV-infected population (40). Furthermore, PLWH with a history of ATS use disorders are more likely to delay ART initiation (41). High-risk sexual behaviors and needle-sharing practices associated with ATS use may also increase susceptibility to sexually transmitted infections and blood-borne pathogens (42), potentially contributing to the higher burden of communicable diseases observed at baseline.

After PSM adjusted for potential covariates, we analyzed immune response patterns among 2,372 PLWH during the first 24 months after ART initiation. However, significant differences remained between the two groups in baseline viral load, the interval between HIV diagnosis and ART initiation, and WHO stage. PLWH with ATS use disorders have lower rates of HIV testing prior to treatment (43), which may also contribute to higher baseline viral load (41). The prolonged diagnostic-to-treatment intervals observed in this population may further exacerbate viral load elevation, potentially accounting for the more advanced WHO stage among ATS-exposed individuals.

The associations of covariates with immune response observed in our study are consistent with prior evidence. Established predictors—including age, BMI, WHO stage, interval between diagnosis and ART initiation, viral load, CD4+ T-cell count, and communicable diseases—remain consistent risk factors for impaired immune recovery in PLWH. A previous cohort study showed that PLWH with ATS use disorders had decreased CD4+ T-cell counts and lower CD4+/CD8+ ratios after ART, which was unfavorable for immune response (44). Our analysis corroborates these observations: PLWH with a history of ATS use disorders showed slower CD4+ T-cell recovery, along with lower cumulative probabilities of immune response. Furthermore, longer historical ATS exposure was associated with reduced immune response rates in PLWH, potentially reflecting progressive immune-system impairment related to the severity of ATS-associated damage.

Although our study is observational, our findings are biologically plausible in light of prior research suggesting that ATS exposure can perturb CD4+ T-cell activation, homeostatic maintenance, and survival, thereby constraining immune reconstitution after ART initiation. First, chronic ATS exposure has been linked to immune dysfunction (16, 17) and may impair T-cell survival and bioenergetic fitness through oxidative stress and mitochondrial injury, potentially limiting sustained homeostatic proliferation during immune recovery. Second, ATS-associated immune activation may dysregulate effective T-cell activation and clonal expansion following antigenic or TCR stimulation, leading to inefficient reconstitution even under virologic suppression (18, 45, 46). Third, ATS-enhanced HIV replication (47, 48) could increase antigenic burden and promote persistent immune activation, a well-recognized driver of incomplete immune reconstitution in HIV. Taken together, this framework provides a mechanistic rationale linking ATS exposure to altered T-cell activation, homeostasis, and survival, and supports the interpretation of impaired immune recovery among ATS-exposed PLWH. Future *in vitro* studies, complemented by targeted immunophenotyping, are warranted to test these pathways directly.

Notably, in the subgroup analysis stratified by baseline CD4+ T-cell count, the association between ATS exposure and immune response in PLWH was no longer statistically significant. We speculate that, in this population, the effect of a low CD4+ T-cell count on immune response may be substantially greater than that of a history of ATS exposure. Previous studies have likewise shown that patients with low baseline CD4+ T-cell counts experience more severe immunosuppression and have greater difficulty achieving immune response (26, 49). However, as the database does not provide detailed information on the specific types of ATS used by PLWH, it was not possible to determine whether methamphetamine, ecstasy, or other ATS exert differential effects on immune response. Future studies could further investigate the impact of specific ATS types on immune response.

Previous studies have suggested that ATS abuse can compromise adherence among PLWH and increase HIV viral load (19–22). Building on this evidence, our study indicates that even after ATS use has ceased, a history of ATS abuse remains a risk factor for achieving immune reconstitution in PLWH. Therefore, for PLWH with a history of ATS abuse, in addition to initiating ART as early as possible and strengthening treatment adherence, emergency management should be integrated into HIV clinics to facilitate CD4+ T-cell count recovery (50, 51).

## Conclusions

A history of ATS use disorders significantly compromises immune response in PLWH after ART initiation, and the cumulative duration of ATS exposure shows a dose-dependent association with suboptimal immunological recovery. These findings highlight the need for clinical interventions that incorporate adjunctive immune-enhancing measures at the time of ART initiation for PLWH with ATS use disorders. Treatment protocols should prioritize optimized ART regimens with greater efficacy, implement comprehensive strategies for opportunistic infection prevention, and integrate specialized clinical services, including drug resistance profiling, to address the distinct therapeutic needs of this population.

## Data Availability

The raw data supporting the conclusions of this article will be made available by the authors, without undue reservation.
