# Supplementary material for: The effect of amphetamine-type stimulants on immune response in HIV-infected individuals: a retrospective cohort study
Source: Front Immunol. 2026 Feb 9;17:1775159. doi: 10.3389/fimmu.2026.1775159 (PMC12926158; doi:10.3389/fimmu.2026.1775159)
Supplement: Supplementary file 1 [file Table1.docx]

**Supplementary materials**

**Table S1**: Data missing and multiple imputation during follow-up process.

|  | **Total Data** | **Missing Data** | **CD4+T-cell count(cells/mm^3^)** | | **P** |
| --- | --- | --- | --- | --- | --- |
|  |  |  | **Before interpolation** | **After interpolation** |  |
| 3rd month | 59216 | 3531 (5.95%) | 203 (147, 278) | 203 (127, 278) | 0.831 |
| 6th month | 59104 | 4357 (7.37%) | 228 (172, 304) | 228 (172, 304) | 0.693 |
| 9th month | 58866 | 5232 (8.89%) | 253 (195, 329) | 253 (195, 329) | 0.854 |
| 12th month | 58610 | 4579 (7.81%) | 277 (213, 354) | 277 (213, 353) | 0.953 |
| 15th month | 58363 | 6582 (11.28%) | 297 (231, 372) | 297 (231, 372) | 0.607 |
| 18th month | 58128 | 6249 (10.75%) | 315 (247, 389) | 315 (247, 389) | 0.649 |
| 21st month | 57903 | 4098 (7.08%) | 332 (261, 404) | 331 (260, 403) | 0.125 |
| 24th month | 57695 | 6743 (11.69%) | 346 (273, 417) | 345 (271, 416) | 0.062 |

**Table S2**: Baseline conditions of exposed and Non-exposed groups before and after PSM

|  | **Before PSM** | | | **After PSM** | | |
| --- | --- | --- | --- | --- | --- | --- |
| **Variable** | **Exposed group**  **(1356)** | **Non-** **exposed group (57960)** | **P** | **Exposed group**  **(1186)** | **Non- exposed group (1186)** | **P** |
| **Gender (male)** | 1166(86%) | 42136(72.7%) | ＜0.001 | 1009(85.1%) | 1031(86.9%) | 0.193 |
| **Age** | 29(21,37) | 38(28,52) | ＜0.001 | 30(21,37) | 26(22,36) | 0.148 |
| **BMI** | 19.9(17.9, 22.5) | 20.9(18.8,23.0) | ＜0.001 | 19.9(17.8,22.4) | 20.3(17.5,22.6) | 0.409 |
| **Time interval^a^** | 60(19,229) | 20(9,65) | ＜0.001 | 44(18-175) | 18(8,55) | ＜0.001 |
| **Viral load** | 62284(14378,  263650) | 69484(21004,  300985) | ＜0.001 | 52027(12588,  225987) | 15082(4767  ,54933) | ＜0.001 |
| **CD4+T cell count** | 155(102,213) | 177(119,246) | ＜0.001 | 161(106,221) | 159(99,221) | 0.643 |
| **WHO** |  |  | ＜0.001 |  |  | 0.009 |
| Ⅰ | 948(69.9%) | 32433(56%) |  | 823(69.7%) | 830(69.9%) |  |
| Ⅱ | 136(10%) | 9889(17.1%) |  | 167(14.1%) | 120(10.1%) |  |
| Ⅲ | 200(14.7%) | 7097(12.2%) |  | 139(11.7%) | 163(13.7%) |  |
| Ⅳ | 72(5.3%) | 8541(14.7%) |  | 57(4.8%) | 73(6.2%) |  |
| **Route of infection** |  |  | ＜0.001 |  |  | 0.535 |
| Heterosexual | 859(63.3%) | 52510(90.6%) |  | 808(68.1%) | 772(65.1%) |  |
| Homosexual | 307(22.6%) | 4299(7.4%) |  | 242(20.4%) | 259(21.8%) |  |
| Blood | 39(2.9%) | 392(0.7%) |  | 29(2.4%) | 29(2.4%) |  |
| Maternal-infant | 12(0.9%) | 69(0.1%) |  | 10(0.8%) | 14(1.2%) |  |
| Unclear | 139(10.3) | 690(1.2) |  | 97(8.2%) | 112(9.4%) |  |
| **Syphilis** | 217(16%) | 7521(13%) | 0.001 | 173(14.6%) | 170(14.3%) | 0.453 |
| **Tuberculosis** | 122(9.0%) | 1104(7.3%) | 0.018 | 104(8.8%) | 118(9.9%) | 0.324 |
| **Hepatitis B** | 149(11%) | 5037(8.7%) | 0.003 | 122(10.3%) | 123(10.4%) | 0.946 |
| **Hepatitis C** | 43(3.2%) | 1104(1.9%) | 0.001 | 34(2.9%) | 40(3.4%) | 0.479 |
| **ART scheme** |  |  | ＜0.001 |  |  | 0.911 |
| EFV+3TC+TDF | 1015(74.9%) | 50026(86.3%) |  | 903(76.1%) | 895(75.5%) |  |
| 3TC+AZT+NVP | 123(9.1%) | 3450(6%) |  | 111(9.4%) | 111(9.4%) |  |
| 3TC+AZT+EFV | 64(4.7%) | 1104(1.9%) |  | 71(6.0%) | 69(5.8%) |  |
| Other | 154(11.4%) | 3380(5.8%) |  | 101(8.5%) | 111(9.4%) |  |

a: The interval between HIV diagnosis and initiation of ART; PSM : propensity scores matching; ATS : amphetamine-type stimulant.

**Table S3**: Deaths of PLWH in the exposed and Non-exposed groups during follow-up

|  | AIDS related-deaths | P | Non AIDS related deaths | P |
| --- | --- | --- | --- | --- |
| ATS group | 53 | 0.001 | 1 | 0.11 |
| Non-ATS group | 24 |  | 9 |  |
